# Dexamethasone pretreatment impairs the thymidylate synthase inhibition mediated flare in thymidine salvage pathway activity in non-small cell lung cancer

**DOI:** 10.1371/journal.pone.0202384

**Published:** 2018-08-24

**Authors:** Xiao Chen, Yizeng Yang, Sharyn I. Katz

**Affiliations:** 1 Department of Radiology, University of Pennsylvania Perelman School of Medicine, Philadelphia, PA, United States of America; 2 Department of Radiology, Institute of Surgery Research, Daping Hospital, Third Military Medical University, Chongqing, China; 3 Department of Medicine, University of Pennsylvania Perelman School of Medicine, Philadelphia, PA, United States of America; University of PECS Medical School, HUNGARY

## Abstract

**Introduction:**

Successful inhibition of thymidylate synthase (TS) by pemetrexed, a TS inhibitor, results in a reproducible transient burst or “flare” in thymidine salvage pathway activity at 2 hrs. of therapy which can be measurable with FLT-PET ([18F]fluorothymidine-positron emission tomography) in non-small cell lung cancer (NSCLC). Routine administration of dexamethasone with pemetrexed-based therapy could potentially confound this imaging approach since dexamethasone is known to inhibit expression of thymidine kinase 1, a key enzyme in the thymidine salvage pathway. Here we examine the potential impact of dexamethasone on the TS inhibition-mediated thymidine salvage pathway “flare” in NSCLC.

**Materials and methods:**

In order to determine NSCLC cell line sensitivity to dexamethasone and pemetrexed, IC_50_ studies were performed on NSCLC cell lines H23, H1975, H460, H1299. TS inhibition-mediated “flare” in thymidine salvage pathway activity was then measured at 2hrs. of exposure to pemetrexed and cisplatin in NSCLC cells lines following using ^3^H-thymidine incorporation assays under the following conditions: control (no chemotherapy or dexamethasone), or treated with pemetrexed and cisplatin without dexamethasone, with 24 hrs. pre-treatment of dexamethasone or with dexamethasone administered together with chemotherapy. These conditions were chosen to model the delivery of pemetrexed-based therapy in the clinic.

**Results:**

The IC_50_ of H23, H1975, H460, H1299 for dexamethasone and pemetrexed were 40, 5.9, 718, 362 μM and 0.22, 0.73, 0.14 and 0.66 μM respectively. Significant blunting of the thymidine salvage pathway “flare” is observed at 2hrs. of pemetrexed-based therapy when dexamethasone sensitive cell lines H23 and H1975 were pretreated with dexamethasone but not when dexamethasone was given together with pemetrexed therapy or in the setting of dexamethasone resistance (H460 and H1299).

**Conclusion:**

24 hr. pretreatment with dexamethasone, but not same day co-administration of dexamethasone with therapy, impairs the TS inhibition-mediated “flare” in thymidine salvage pathway activity in NSCLC.

## Introduction

Therapeutic inhibition of thymidylate synthase (TS) is a commonly used strategy in management of a number of malignancies including non-small cell lung cancer (NSCLC). The successful inhibition with commonly used therapeutics, 5-FU[1–3], pemetrexed[4, 5] and capecitabine[6] have all been demonstrated to induce a transient burst of compensatory activity through the thymidine kinase pathway within hours of exposure to the chemotherapeutic. This transient burst in thymidine salvage pathway activity is measureable with FLT-PET ([18F]fluorothymidine -position emission tomography)[3–6]. FLT, first described by Shields *et al[7]*, is an analog of thymidine and highly specific surrogate marker of proliferation, including in lung cancer[8–11].

In previous work by our laboratory, we have demonstrated that pemetrexed-induced inhibition of TS results in a “flare” in thymidine salvage pathway activity observable at 2 hrs. of therapy *in vitro* by ^3^H-thymidine incorporation assays and *in vivo* using FLT-PET in a mouse model of NSCLC and in human patients[4]. This increase pemetrexed-induced “flare” in thymidine salvage pathway activity appeared to be on the basis of an increase in thymidine kinase 1 (TK1) activity[1, 4] and mobilization of equilibrative nucleoside transporter 1 (ENT1) from around the nucleus to the cellular membrane[2, 4]. Furthermore, the pemetrexed-induced “flare” appeared to be predictive of NSCLC sensitivity both *in vitro* and *in vivo*. This preclinical work suggests a potential role for FLT-PET imaging of TS inhibition as a means of early detection of NSCLC sensitivity to pemetrexed which could be a useful tool in cancer management if translated to the clinic, though one exploratory clinical trial of pemetrexed therapy in non-small cell lung cancer[12] did not see a correlation between the pemetrexed-induced “flare” and eventual therapy success.

An important potential caveat to the translation of this PET imaging strategy, potentially underlying the findings by Frings *et al[12]*, is the fact that combination therapy with pemetrexed and cisplatin or carboplatin is often administered with dexamethasone, which can itself inhibit proliferation[13–16]. Dexamethasone is administered primarily to mitigate untoward side effects often experienced with this chemotherapeutic combination including hypersensitivity reactions and fluid retention reactions[17]. Typically dexamethasone is administered with a 3 day regimen, dosed the day before, day of and day after the administration of pemetrexed and cisplatin or carboplatin, although some oncologists choose to only give dexamethasone on the day of therapy[17, 18]. Since dexamethasone has been demonstrated to suppress TK1 expression[14], and TK1 activity and expression is central to the pemetrexed-induced “flare” in thymidine salvage pathway activity, we asked the question whether co-administration of dexamethasone with pemetrexed could impair the use of the pemetrexed-induced “flare” as a predictive imaging tool in this setting. Here we examine the impact of dexamethasone on the pemetrexed-induced “flare” in human NSCLC cell lines in the setting of co-administration of dexamethasone.

## Materials and methods

### Chemotherapeutics and imaging radiopharmaceuticals

For *in vitro* studies, as we have previously described in detail[4, 5], pemetrexed (Santa Cruz Biotechnology, Dallas, Texas) and cisplatin (Sigma-Aldrich Corp., St. Louis, MO) were purchased in their solid form and stored at -20°C as 0.2 mM and 1 mM stocks (diluted in sterile water) respectively. Dexamethasone (Sigma-Aldrich Corp., St. Louis, MO) was also purchased in solid form and stored at -20°C as 2.5 mM stocks (diluted in 100% ethanol).

### Cell lines and culture

As we have previously described in detail[4, 5], all of the human non-small cell lung cancer cell lines used in this study (H23, H1975, A549, H460, H1299) were obtained from American Type Culture Collection (ATCC, Manassas, VA). These NSCLC cell lines were cultured in RPMI medium containing 10% fetal bovine serum (FBS), 100 IU/mL penicillin, and 100 μg/mL streptomycin in a humidified incubator in 5% CO2 at 37°C. in order to passage the cell lines, cells were first detached from the culture plate surface using sterile 0.05% trypsin-EDTA solution then passaged at a 1:3 dilution.

### IC_50_ calculations

The IC_50_ for each cell line for each cisplatin, pemetrexed and dexamethasone were performed as we have previously described in detail[5]. Cultured NSCLC cell lines (H23, H1975, A549, H460, or H1299) were harvested and seeded into a 24-well plate (2 X 10^4^ cells per well) in RPMI1640 culture medium and incubated for 24 hours at 37°C in a 5% CO_2_ incubator. The culture medium was then replaced with 100 μL of fresh medium containing varying concentrations of dexamethasone, cisplatin or pemetrexed (0, 0.01, 0.1, 1, 10, 100 μM) and incubated for 72 hours at 37°C in a 5% CO_2_ incubator. The IC_50_ assay was performed then performed using the MTT Cell Growth Assay Kit (Sigma-Aldrich, St. Louis, MO). Absorbance of the converted dye was measured using a Beckman DU-600 Spectrophotometer (Beckman Coulter Life Sciences, Indianapolis, IN) and data analyzed using the statistical software SPSS 19.0 (IBM, Chicago, USA).

### ^3^H-thymidine assays

[^3^H]-thymidine assays were performed as we have previously described in detail[4, 5]. [^3^H]-thymidine (Perkin Elmer NET355001MC, PerkinElmer, Waltham, MA) was utilized for *in vitro* assessment of therapy-induced changes in thymidine salvage pathway activity in cultured human NSCLC cells. [^3^H]-thymidine specific activity was >10Ci(370GBq)/mmol and radiochemical purity >97%. H23, H1975, A549, H460, or H1299 cells were seeded (1×10^6^/well) in 6-well plate in RPMI1640 supplemented with 10% FBS and antibiotics, incubated 24 hours in 5% CO_2_ at 37°C. When cell cultures achieved 80% confluence, cells were exposed to treatment with one of the following treatment groups: vehicle (sterile water); dexamethasone (100 nM); combination of pemetrexed (100 nM) and cisplatin (10 μM); combination of pemetrexed (100 nM) and cisplatin (10 μM) with 24hrs. of dexamethasone (100 nM) pretreatment; and combination of pemetrexed (100 nM) and cisplatin (10 μM) with dexamethasone given concurrently. Cells were exposed to these treatment exposures in growth media for varying exposure times ranging up to 48 hours. Drug-containing medium was then removed, and the cells were then washed and pulsed with 5 μCi [^3^H]-thymidine/well for 1 hour. The cells were then washed and scraped into plastic vials. Scintillant (10 mL; Research Products International Corp., Mount Prospect, IL) was added to each vial and the radioactivity was counted on a scintillation counter (Beckman Coulter LS6500, Beckman Coulter Life Sciences, Indianapolis, IN).

## Results

### IC_50_ analysis of NSCLC cell lines

The IC_50_ calculations (**Table 1**) for H23 and H1975 were 40 μM and 5.9 μM respectively for dexamethasone and 0.22 μM and 0.73 μM respectively for pemetrexed. The IC_50_ calculations for H460 and H1299 were 718 μM and 362 μM respectively for dexamethasone and 0.14 μM, 0.66 μM respectively for pemetrexed. All 4 cell lines demonstrated sensitivity to cisplatin which is included since these *in vitro* experiments model clinical administration of pemetrexed-based therapy.

**Table 1:** IC_50_ calculations for human NSCLC cell lines.

Cell lines	Dexamethasone (μM)	Pemetrexed (μM)	Cisplatin (μM)
H23	40.04	0.22	0.67
H1975	5.90	0.73	6.71
H460	718.15	0.14	3.22
H1299	362.46	0.66	7.04

### Thymidylate synthase inhibition mediated “flare” in thymidine salvage pathway is blunted by pretreatment with dexamethasone

**Fig 1:**
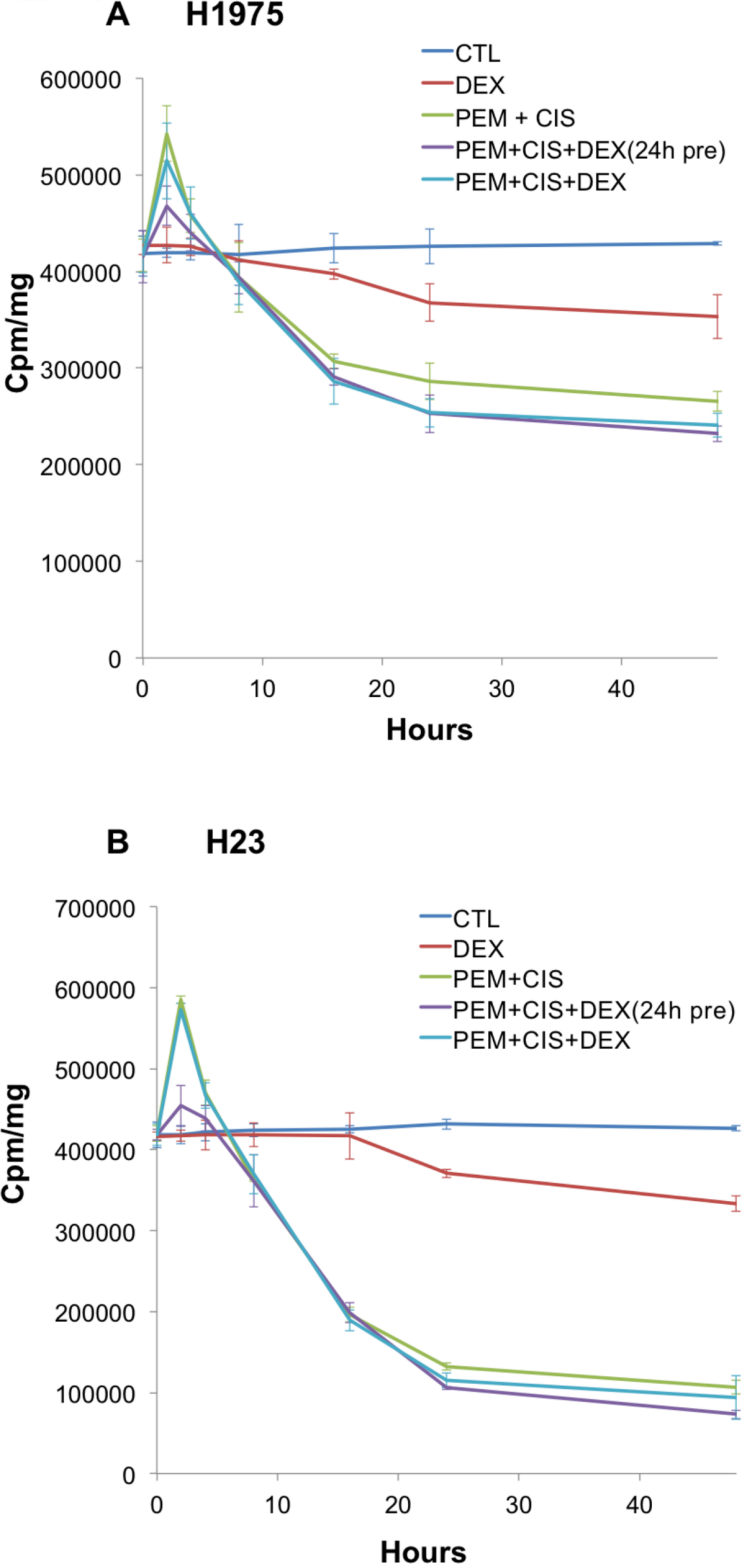
Pretreatment with dexamethasone results in blunting of the pemetrexed-induced thymidine salvage pathway “flare” in dexamethasone sensitive lines. Thymidine salvage pathway activity of the human NSCLC cell lines was assessed using ^3^H-thymidine incorporation assays. Dexamethasone sensitive cell lines, H1975 and H23 revealed blunting of the thymidine salvage pathway “flare” when cells were pretreated with dexamethasone for 24hrs. No effect on the pemetrexed-induced “flare” was observed in these cell lines when dexamethasone was given concurrently with chemotherapy (without pretreatment). A: H1975. B: H23.

**Fig 2:**
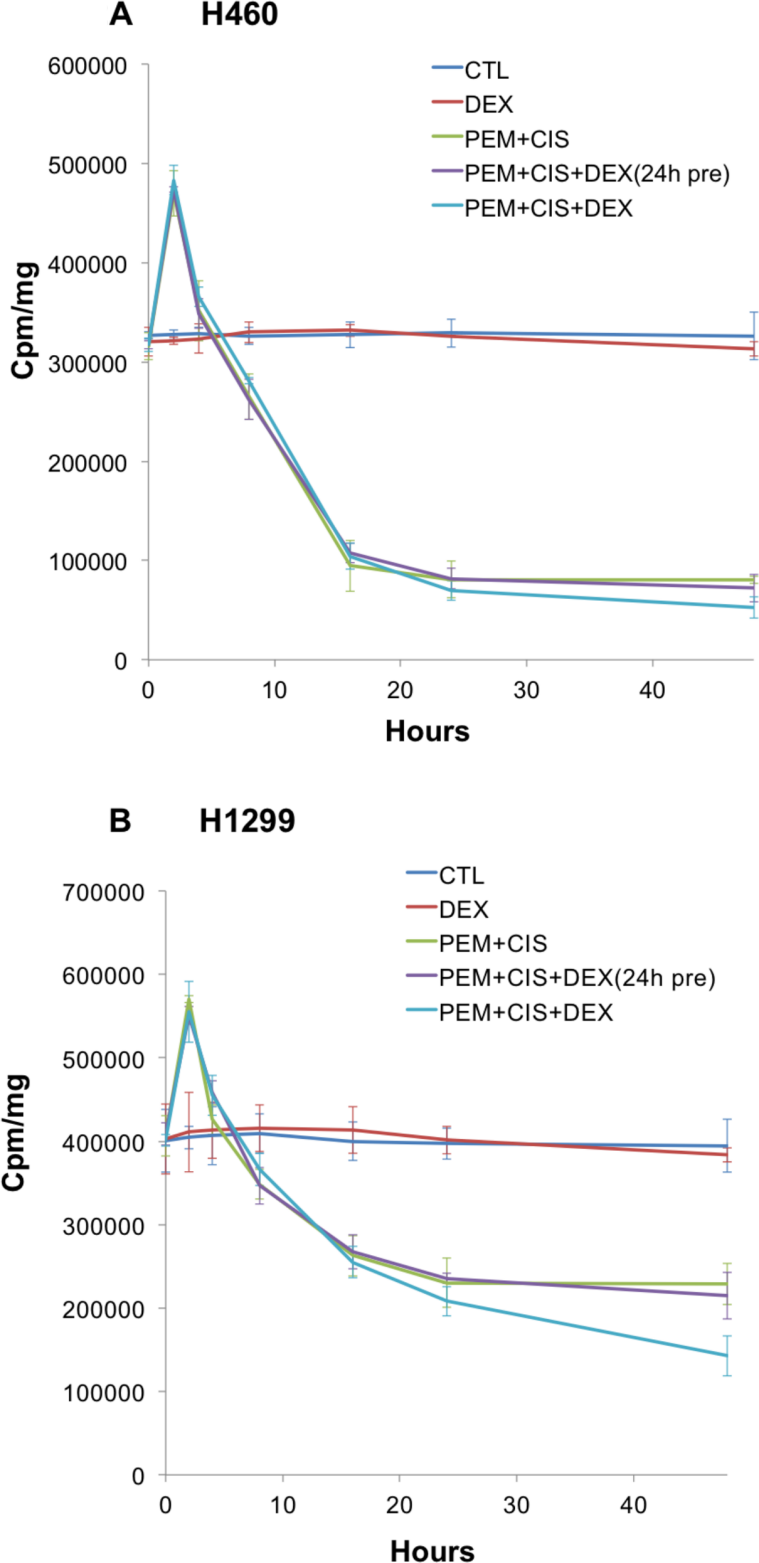
No impact of dexamethasone pre-treatment is observed on the pemetrexed-induced thymidine salvage pathway “flare” in dexamethasone resistant cell lines. Thymidine salvage pathway activity of the human NSCLC cell lines was assessed using ^3^H-thymidine incorporation assays. Dexamethasone resistant cell lines, H460 and H1299 do not reveal evidence of blunting of the thymidine salvage pathway “flare” when cells were either pretreated with dexamethasone for 24hrs. or dexamethasone was given concurrently with chemotherapy. A: H460. B: H1299.

All four pemetrexed sensitive cell lines, H23, H1975, H460 and H1299 reveal a pemetrexed-induced “flare” in thymidine salvage pathway activity at 2 hrs. of therapy **(Figs 1 and 2)** similar to what we have previously reported[4, 5]. Significant blunting of the thymidine salvage pathway “flare” is observed at 2hrs. of pemetrexed-based therapy when dexamethasone sensitive cell lines H23 and H1975 were pretreated with dexamethasone **(Fig 1A and 1B)**. This blunting of the pemetrexed-induced “flare” is not observed when dexamethasone was given together with pemetrexed therapy or in dexamethasone resistant cell lines H460 and H1299 **(Fig 2A and 2B)**.

## Discussion

Published work in our laboratory and others have demonstrated that successful TS inhibition can result in a compensatory “flare” in thymidine salvage pathway activity that occurs at approximately 2 hrs. following the start of therapy both *in vitro[1–5, 19]* and *in vivo[3–6, 20, 21]*. This effect was also observed *in vivo* both in a mouse model of human NSCLC and in a human patient with NSCLC using FLT-PET ([18F]fluorothymidine-positron emission tomography) imaging[4]. The pemetrexed-induced “flare” also appears predictive of NSCLC sensitivity to pemetrexed[5] raising the possibility that FLT-PET may be a useful imaging tool for determining successful TS inhibition in NSCLC patients. It is important to note that an exploratory clinical trial of the FLT measured pemetrexed-induced flare in non-small cell lung patients receiving pemetrexed by Frings *et al* did not demonstrate a correlation between the FLT “flare” and eventual tumor response to therapy. This may be due, in part, to the fact that the imaging of the TS inhibition-induced “flare” measures success of TS inhibition but does not measure tumor cell sensitivity to cisplatin. Their work underscores the need for further study of this imaging strategy prior to clinical translation.

Another potential confounder for clinical translation of the FLT imaging “flare” technique for assessment of NSCLC sensitivity to pemetrexed is the common co-administration of dexamethasone with pemetrexed-based therapy. Dexamethasone is typically administered along with pemetrexed-based therapy in the setting of NSCLC to prevent untoward side effects of chemotherapy. This could present a potential barrier to translation to the clinic since exposure to dexamethasone is known to decrease expression of TK1[14, 22], and TK1 activity plays a central role in mediating the TS inhibition-induced thymidine salvage pathway “flare”. In many clinical settings, dexamethasone is administered with a 3-day regimen beginning the day before administration of pemetrexed-based therapy, although some oncologists choose to administer dexamethasone only on the day of therapy. Here we demonstrate that the pemetrexed-induced “flare” in the thymidine salvage pathway is mitigated by pretreatment with dexamethasone for 24 hrs. but does not appear impacted by administration of dexamethasone concurrently with pemetrexed.

The data presented here suggests that imaging of TS-inhibition with FLT-PET may be preferred in settings where premedication with dexamethasone on the day before pemetrexed-based therapy can be omitted. Our findings are supported by similar work recently published by McHugh *et al[16]* who demonstrated that by 24 hrs. of treatment with dexamethasone, there is a significantly decreased cellular retention of FLT and abolishment of the pemetrexed-induced FLT “flare” in non-small cell lung cancer *in vitro* and *in vivo*. Our work presented in this manuscript also demonstrates a similar reduction of the pemetrexed-induced thymidine salvage pathway “flare” when preceded by 24 hr. pre-treatment with dexamethasone. In addition, here we also provide evidence that co-administration of dexamethasone with chemotherapeutics on the day of therapy does not impair the pemetrexed-induced “flare” in thymidine salvage pathway activity. This is important in suggesting a means of translating this imaging strategy for assessment of TS inhibition without the confounding effects from pre-treatment with dexamethasone. Further carefully controlled clinical trials are needed to determine if the FLT-PET “flare” can be successful in the early assessment of TS-inhibition in the clinical setting of pemetrexed-based therapy for NSCLC.
